# Disease-specific mortality among stage I–III colorectal cancer patients with diabetes: a large population-based analysis

**DOI:** 10.1007/s00125-012-2555-8

**Published:** 2012-04-24

**Authors:** L. V. van de Poll-Franse, H. R. Haak, J. W. W. Coebergh, M. L. G. Janssen-Heijnen, V. E. P. P. Lemmens

**Affiliations:** 1 Comprehensive Cancer Centre South (CCCS)/Eindhoven Cancer Registry, PO Box 231, 5600 AE Eindhoven, the Netherlands; 2 CoRPS—Center of Research on Psychology in Somatic Diseases, Department of Medical Psychology, Tilburg University, Tilburg, the Netherlands; 3 Department of Internal Medicine, Maxima Medical Centre, Eindhoven, the Netherlands; 4 Department of Public Health, Erasmus Medical Centre, Rotterdam, the Netherlands; 5 Department of Clinical Epidemiology, Viecuri Medical Centre, Venlo, the Netherlands

**Keywords:** Colorectal cancer, Diabetes, Disease-specific mortality, Population-based

## Abstract

**Aims/hypothesis:**

The aim of our study was to investigate overall and disease-specific mortality of colorectal cancer patients with diabetes.

**Methods:**

In this population-based study, we included all colorectal cancer patients, newly diagnosed with stage I–III cancer, between 1997 and 2007 in the registration area of the Eindhoven Cancer Registry. Stage of cancer, cancer treatment and comorbidities were actively collected by reviewing hospital medical records. Data on patients with and without diabetes were linked to Statistics Netherlands to assess vitality, date of death and underlying cause of death. Follow-up of all patients was completed until 1 January 2009.

**Results:**

We included 6,974 patients with colon cancer and 3,888 patients with rectal cancer, of whom 820 (12%) and 404 (10%), respectively, had diabetes at the time of cancer diagnosis. During follow-up, death occurred in 611 (50%) of 1,224 cancer patients with diabetes and 3,817 (40%) of 9,638 cancer patients without diabetes. Multivariate Cox regression analyses, adjusted for age, sex, socioeconomic status, stage, lymph nodes examined, adjuvant therapy and year of diagnosis, showed that overall mortality was significantly higher for colon (HR 1.12, 95% CI 1.01, 1.25) and rectal (HR 1.21, 95% CI 1.03, 1.41) cancer patients with diabetes than for those without. Disease-specific mortality was only significantly increased for rectal cancer patients (HR 1.30, 95% CI 1.06, 1.60).

**Conclusions/interpretation:**

Diabetes at the time of rectal cancer diagnosis was independently associated with an increased risk of colorectal cancer mortality compared with no diabetes, suggesting a specific interaction between diabetes and rectal cancer. Future in-depth studies including detailed diabetes- and cancer-related variables should elucidate pathways.

## Introduction

The number of older people with cancer is increasing considerably, resulting in a growing proportion of patients who present with multiple coexisting medical conditions [1]. Worldwide, a strongly increasing prevalence of diabetes has been observed [2–4]. Breast, colorectal, endometrial, pancreatic and kidney cancer are more prevalent in patients with diabetes than in those without diabetes. Furthermore, the prevalence of diabetes in these patients is also higher than in other cancer patients or patients without cancer [5]. It is estimated that the number of newly diagnosed cancer patients with diabetes in the Netherlands will double from 5,500 in 2000 to 10,400 in 2015 [6].

Since 1993, the Eindhoven Cancer Registry (ECR) has registered the prevalence of comorbid conditions at the time of cancer diagnosis. About 60% of all newly diagnosed cancer patients older than 65 years had at least one other serious disease, of which diabetes was one of the most common (16%) [1]. In a population-based analysis of all 58,498 cancer patients diagnosed between 1995 and 2002 in the southern Netherlands, we showed that patients with pre-existing diabetes had a significantly increased overall mortality [5]. This result was confirmed by a meta-analysis that included our paper and 22 other papers [7], and a review that focused on colorectal cancer [8].

In 2010, the American Diabetes Association and American Cancer Society reviewed the state of science concerning diabetes and cancer [9, 10]. One of the key issues was a better understanding of whether diabetes influences cancer prognosis above and beyond the prognosis conferred by each disease state independently. Today, only a few studies have evaluated disease-specific mortality among cancer patients with diabetes. Poorly controlled, pre-existing diabetes mellitus has been associated with an increased risk of death attributable to colorectal cancer [11]. Similarly, hyperinsulinaemia and five other previously established components of metabolic syndrome were shown to be prospective risk factors for deaths that can be ascribed to prostate cancer [12]. Furthermore, an increased risk was also observed for endometrial cancer mortality among women with diabetes [13]. Recently, the large prospective Cancer Prevention Study-II Nutrition Cohort showed increased colorectal cancer mortality among patients with colorectal cancer and diabetes [14]. However, other studies found no association between diabetes- and cancer-specific mortality [15–18]. These conflicting results may be explained by small numbers of diabetes patients, a limited number of deaths, and/or no information about cancer treatment [11–13, 15–18].

The aim of our study was to investigate the overall and disease-specific mortality of colorectal cancer patients with diabetes, taking into account differences in age, comorbidity (especially cardiovascular diseases [CVDs]), stage and treatment according to diabetes status. In view of the variable associations between diabetes and cancer risk at specific sites [9, 10, 19], we analysed our data for colon and rectal cancer separately.

## Methods

### Patients and methods

The ECR records data on all patients newly diagnosed with cancer in the southern part of the Netherlands, an area with 2.3 million inhabitants, ten general hospitals and two large radiotherapy institutes. Trained registration clerks actively collect data on diagnosis and staging (tumour, nodes, metastasis [TNM]) and detailed information about initial treatment from hospital medical records. Registration takes place 6–12 months after diagnosis. By means of an independent case ascertainment method, the completeness of the registration has been estimated to exceed 95% [20]. The medical record is generally regarded as the most complete source of information on the patient’s past and current health status [21]. Since 1993, serious comorbidity with prognostic impact has been recorded for all patients. A slightly modified version of the widely used Charlson comorbidity index [22] is used [1]. Comorbidity was defined as life-shortening diseases that were present at the time of cancer diagnosis. Drug use, often recorded from general practitioners’ letters, served as an indicator of active disease. Diabetes mellitus included both type I and type II disease and was registered as a dichotomous variable (yes/no), as were all other concomitant conditions. CVD included myocardial infarction, cardiac insufficiency, angina pectoris, coronary artery bypass graft, peripheral arterial disease, and cerebrovascular diseases. Comorbidity data since 1995 are considered accurate, i.e. complete and correct.

We included all colorectal cancer (International Classification of Diseases for Oncology codes C18.0–C20.9) patients, newly diagnosed with stage I–III cancer, between 1997 and 2007. We excluded patients for whom the site of the primary tumour within the colorectum was not known (1.5% of total). Oncological treatment was defined as surgery, radiotherapy and/or chemotherapy. Surgery did not comprise diagnostic operations. Data on patients with colorectal cancer with or without diabetes from the population-based ECR were linked to mortality data from the database of Statistics Netherlands.

Statistics Netherlands registers vital status, including, if applicable, date of death of a person as recorded by the municipal population registries. Statistics Netherlands also documents the primary underlying cause of death (UCOD) as recorded by the attending physician. This is performed according to the World Health Organization UCOD definition ‘the disease or injury which initiated the chain of morbid events leading directly to death, or the circumstances of the accident or violence which produced the fatal injury’ and in accordance with the rules of the International Classification of Diseases-tenth revision. Follow-up of all patients was completed until 1 January 2009.

Postal codes of residential areas are used to establish the socioeconomic status (SES) of diagnosed cancer patients, also provided by Statistics Netherlands [23]. At the six-position level of postal code, data on household income and economical value of the house are available from fiscal data. This information was transformed into four categories: low, medium and high SES and patients who were institutionalised (such as in a nursing home). ECR and Statistics Netherlands data were linked by use of postal codes and dates of birth. If patients could not be found in Statistics Netherlands with this strategy, date of death was also provided by the ECR to track the patient in Statistics Netherlands files.

### Statistical analysis

The SAS computer package (version 9.1 for Windows; SAS Institute, Cary, NC, USA) was used for all statistical analyses. All analyses were performed separately for colon and rectal cancer patients. Differences in patient, tumour and treatment characteristics according to diabetes status were tested by *χ* ^2^ analysis or *t* test, as appropriate. Non-parametric equivalents were applied when normality and homogeneity assumptions were violated. Survival analysis was carried out using the life table method to evaluate the prognosis after diagnosis for cancer patients with or without diabetes. Survival time was defined as the time from diagnosis to death or 1 January 2009 for the patients who were still alive. Five-year cumulative survival probability and its standard error were calculated for those with or without diabetes and compared by means of the logrank test.

The independent prognostic effect of diabetes on overall mortality and colorectal cancer-specific mortality was estimated using Cox proportional hazards regression models. The proportional hazard assumption of diabetes was evaluated by estimating Kaplan–Meier curves. The effect of diabetes over time satisfied the assumption of proportionality as the graphs of the log (log[survival]) versus log of survival time exhibited parallel lines for the groups of diabetic and non-diabetic patients. Confounding variables included for adjustment in the Cox proportional hazards regression models were determined a priori [24]: age at diagnosis, sex, stage, number of examined lymph nodes (≥10), adjuvant therapy (chemotherapy or radiotherapy), SES, year of diagnosis, hypertension, CVD, cerebrovascular disease, previous cancer and lung disease. Effect modification between diabetes and all potential confounding variables was assessed by adding interaction terms diabetes × confounding variable in the multivariate Cox proportional hazards regression models. HRs with 95% CIs and *p* values are reported.

## Results

**Fig. 1:**
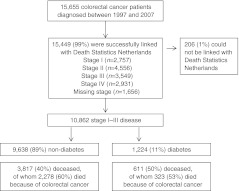
Flow chart of patient selection

Between 1997 and 2007, 15,655 colorectal cancer patients were diagnosed in the ECR area (Fig. 1). Of these, 15,449 (99%) were successfully linked with Death Statistics Netherlands. After exclusion of patients with stage IV disease or missing information on stage, we included 6,974 colon cancer and 3,888 rectal cancer patients with stage I–III disease, of whom 820 (12%) and 404 (10%), respectively, had diabetes at cancer diagnosis. Colon cancer patients with diabetes were on average 4 years older than their counterparts without diabetes, had a lower SES, more additional comorbid conditions and less often received adjuvant systemic therapy (Table 1). Colon cancer patients with diabetes had more often been diagnosed with a previous cancer, hypertension, CVD, cerebrovascular accident (CVA) and lung disease than those without diabetes. There were no differences in sex, stage, grade and number of lymph nodes examined. Although not statistically significant, colon cancer patients with diabetes appeared to be diagnosed more often in more recent periods than those without diabetes. Rectal cancer patients with diabetes were on average 5 years older than their counterparts without diabetes, and had a lower SES and more additional comorbid conditions (Table 2). Rectal cancer patients with diabetes also had been diagnosed more often with a previous cancer, hypertension, CVD, CVA and lung disease. There were no differences in the presence of diabetes with respect to sex, stage, grade, number of examined lymph nodes, and receipt of adjuvant therapy or period of diagnosis.

**Table 1:** Characteristics of patients with colon cancer stage I–III according to diabetes status

Characteristic	Diabetes (*n* = 820)	No diabetes (*n* = 6,154)	*p* value
Male	376 (46)	3,119 (51)	0.0094
Age (years)	73.0 ± 8.8	68.8 ± 11.1	<0.0001
Age			
0–59	66 (8)	1,218 (20)	
60–69	191 (23)	1,707 (28)	
70–79	354 (43)	2,201 (36)	
80+	209 (25)	1,028 (17)	<0.0001
Stage			
I	150 (18)	1,221 (20)	
II	409 (50)	2,849 (46)	
III	261 (32)	2,084 (34)	0.15
Examined lymph nodes			
Median (25–75%)	7 (3–11)	7 (4–11)	
≥10	197 (32)	1,544 (33)	0.48
Therapy			
Surgery alone	677 (83)	4,715 (77)	
Surgery and systemic	118 (14)	1,257 (20)	
Other/unknown	25 (3)	182 (3)	0.0008
SES			
Low	291 (36)	1,610 (27)	
Medium	276 (34)	2,305 (38)	
High	174 (22)	1,819 (30)	
Institutionalised	68 (8)	296 (5)	<0.0001
Comorbidity, excluding diabetes			
0	163 (20)	2,098 (34)	
1	285 (35)	1,946 (32)	
2 or more	372 (45)	1,450 (24)	
Missing	0 (−)	660 (11)	<0.0001
Type of comorbidity			
Previous cancer	135 (16)	822 (13)	0.015
Hypertension	365 (45)	1,242 (20)	<0.0001
CVD	344 (42)	1,441 (23)	<0.0001
CVA	80 (10)	239 (4)	<0.0001
Lung diseases	101 (12)	557 (9)	0.003
Period of cancer diagnosis			
1997–1999	176 (21)	1,401 (23)	
2000–2002	200 (24)	1,721 (28)	
2003–2005	255 (31)	1,746 (28)	
2006–2007	189 (23)	1,286 (21)	0.061

Unless otherwise stated, values are *n* (%) or mean ± SD

**Table 2:** Characteristics of patients with rectal cancer stage I–III according to diabetes status

Characteristic	Diabetes (*n* = 404)	No diabetes (*n* = 3,484)	*p* value
Male	230 (57)	2,081 (57)	0.28
Age (years)	71.2 ± 8.4	66.1 ± 11.2	<0.0001
Age			
0–59	42 (10)	964 (28)	
60–69	121 (30)	1,119 (32)	
70–79	173 (43)	1,004 (29)	
80+	68 (17)	397 (11)	<0.0001
Stage			
I	133 (33)	1,253 (36)	
II	142 (35)	1,156 (33)	
III	129 (32)	1,075 (31)	0.47
Examined lymph nodes			
Median (25–75%)	5 (2–8)	5 (2–9)	
≥10	68 (21)	529 (20)	0.52
Therapy			
Surgery alone	152 (38)	1,156 (33)	
Surgery + radiotherapy	183 (45)	1,647 (47)	
Surgery + radiotherapy + systemic	40 (10)	408 (12)	
Surgery + systemic	14 (3)	146 (4)	
Other/unknown	15 (3)	127 (4)	0.23
SES			
Low	134 (34)	794 (23)	
Medium	152 (38)	1,364 (40)	
High	88 (22)	1,111 (33)	
Institutionalised	26 (7)	139 (4)	<0.0001
Comorbidity, excluding diabetes			
0	79 (19)	1,436 (41)	
1	152 (38)	1,060 (30)	
2 or more	173 (43)	634 (18)	
Missing	0 (−)	660 (11)	<0.0001
Type of comorbidity			
Previous cancer	67 (17)	344 (10)	<0.0001
Hypertension	195 (48)	669 (19)	<0.0001
CVD	154 (38)	650 (19)	<0.0001
CVA	34 (8)	105 (3)	<0.0001
Lung diseases	46 (11)	294 (8)	0.047
Period of cancer diagnosis			
1997–1999	79 (20)	781 (22)	
2000–2002	100 (25)	927 (27)	
2003–2005	134 (33)	1,065 (31)	
2006–2007	91 (23)	711 (20)	0.33

Unless otherwise stated, values are *n* (%) or mean ± SD

Of all 10,862 patients diagnosed in the period 1997–2007, 4,428 (41%) deaths were registered up to the follow-up date 1 January 2009 (Table 3). Evaluation of UCOD showed that colon cancer patients most often died of colon cancer (52% of those without diabetes vs 44% with diabetes), whereas in 2% of the patients, rectal cancer was registered as the UCOD. Of the patients diagnosed with rectal cancer, 23% had colon cancer registered as their UCOD. As misclassification of cause of death may have played a role here, we decided to investigate colorectal cancer death as one group in patients diagnosed with colon or rectal cancer. Therefore, throughout this paper, disease-specific survival or mortality always includes both colon and rectal cancer as the primary cause of death or underlying complication. Cancer patients with diabetes more often had diabetes registered as UCOD (4%) or diseases of the circulatory system (18%) compared with cancer patients without diabetes (0% and 12%, respectively).

**Table 3:** Number and percentages of patients, deaths, causes of death and survival according to cancer subsite and diabetes status

Characteristic	Colon		Rectal	
	Diabetes	No diabetes	Diabetes	No diabetes
Number of patients	820	6,154	404	3,484
Number of deaths	417 (51)	2,525 (41)	194 (48)	1,292 (37)
UCOD				
CRC	194 (47)	1,376 (55)	110 (57)	759 (59)
Colon cancer^a^	184 (44)	1,313 (52)	45 (23)	301 (23)
Rectal cancer^b^	10 (2)	63 (2)	65 (34)	458 (35)
NOS digestive cancer	24 (6)	159 (6)	8 (4)	84 (7)
Other cancers	42 (10)	292 (12)	13 (7)	126 (10)
Diabetes	20 (5)	7 (0.3)	5 (3)	3 (0.2)
Circulatory system	75 (18)	332 (13)	36 (19)	138 (11)
Respiratory disease	21 (5)	98 (4)	7 (4)	58 (4)
External causes	4 (1)	26 (1)	1 (0.5)	16 (1)
Infections	3 (0.7)	23 (1)	1 (0.5)	17 (1)
Other	34 (8)	212 (8)	13 (7)	91 (7)
Survival				
5-year overall	51	60	50	64
10-year overall	22	22	31	48
5-year CRC-specific	73	75	67	75
10-year CRC-specific	61	69	60	70

Values are *n* (%) or %

CRC, colorectal cancer; NOS, not otherwise specified

^a^Colon cancer as underlying cause of death

^b^Rectal cancer as underlying cause of death

Compared with patients without diabetes, 5-year overall survival was 9% lower among colon cancer patients and 14% lower among rectal cancer patients with diabetes. The 10-year overall survival rates were 22% and 17% lower, respectively. Differences in cancer-specific 5- and 10-year survival were somewhat smaller (2–9%).

**Fig. 2:**
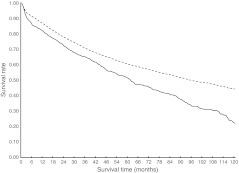
Overall survival for stage I–III colon cancer patients with (solid line) or without (dashed line) diabetes

**Fig. 3:**
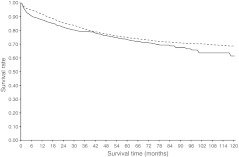
Colorectal cancer-specific survival for stage I–III colon cancer patients with (solid line) or without (dashed line) diabetes

**Fig. 4:**
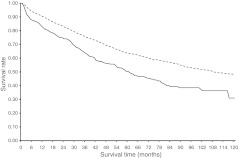
Overall survival for stage I–III rectal cancer patients with (solid line) or without (dashed line) diabetes

**Fig. 5:**
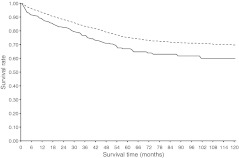
Colorectal cancer-specific survival for stage I–III rectal cancer patients with (solid line) or without (dashed line) diabetes

Unadjusted analyses revealed that the overall survival of cancer patients without diabetes was significantly higher than survival of those with diabetes (Figs 2, 3, 4 and 5). For colon and rectal cancer, the observed unadjusted HR for the effect of diabetes on overall mortality was 1.42 (95% CI 1.28, 1.57) and 1.58 (95% CI 1.36, 1.84), respectively. Multivariate Cox proportional hazards regression analyses, with adjustment for differences in age, sex, SES, stage, number of lymph nodes examined, receipt of adjuvant therapy and period of diagnosis, showed that overall mortality remained significantly higher for colon (HR 1.22, 95% CI 1.09, 1.34) and rectal (HR 1.29, 95% CI 1.11, 1.50) cancer patients with diabetes (data not shown). Bivariate analyses showed that age, sex and therapy were the strongest confounding variables that resulted in a dilution of the effect of diabetes on overall mortality. Further adjustment for hypertension, CVD, cerebrovascular disease, previous cancer and lung disease lowered the HR for overall mortality even further in colon (HR 1.12, 95% CI 1.01, 1.25; Table 4) and rectal (HR 1.21, 95% CI 1.03, 1.41; Table 5) cancer patients with diabetes. CVD was the strongest additional confounding factor in this step, although diabetes remained an independent predictor of overall mortality in colon and rectal cancer patients.

**Table 4:** Multivariate Cox regression analyses of the effect of diabetes on overall and colorectal cancer-specific mortality in stage I–III colon cancer

Characteristic	Overall mortality	Colorectal cancer mortality
Diabetes (vs no)	1.12 (1.01, 1.25)*	1.05 (0.90, 1.23)
Male sex (vs female sex)	1.22 (1.13, 1.31)**	1.12 (1.01, 1.24)*
Age (per 1 year increase)	1.04 (1.04, 1.05)**	1.02 (1.01, 1.03)**
Period of cancer diagnosis		
1997–1999	1	1
2000–2002	0.99 (0.89, 1.10)	0.89 (0.77, 1.03)
2003–2005	0.93 (0.82, 1.04)	0.91 (0.77, 1.06)
2006–2007	0.80 (0.68, 0.94)**	0.86 (0.70, 1.06)
Stage		
I	1	1
II	1.64 (1.46, 1.83)**	2.60 (2.14, 3.17)**
III	3.17 (2.80, 3.60)**	6.83 (5.56, 8.39)**
Examined lymph nodes ≥10 (vs no)	0.69 (0.62, 0.77)**	0.62 (0.53, 0.71)**
Systemic therapy (vs no)	0.68 (0.61, 0.77)**	0.69 (0.59, 0.80)**
SES		
Low	1	1
Medium	0.94 (0.86, 1.03)	0.98 (0.87, 1.11)
High	0.87 (0.79, 0.96)**	0.91 (0.70, 1.05)
Institutionalised	1.09 (0.94, 1.27)	0.96 (0.77, 1.20)
Hypertension (vs no)	0.97 (0.89, 1.06)	0.89 (0.79, 1.01)
CVD (vs no)	1.33 (1.23, 1.45)**	1.12 (0.99, 1.26)
CVA (vs no)	1.32 (1.14, 1.53)**	1.21 (0.97, 1.50)
Previous cancer (vs no)	1.32 (1.20, 1.46)**	1.26 (1.10, 1.45)**
Lung disease (vs no)	1.16 (1.04, 1.30)**	1.09 (0.93, 1.29)

Values are HR (95% CI)

*p < 0.05

**p < 0.01

**Table 5:** Multivariate Cox regression analyses of the effect of diabetes on overall and colorectal cancer-specific mortality in stage I–III rectal cancer

Characteristic	Overall mortality	Colorectal cancer mortality
Diabetes (vs no)	1.21 (1.03, 1.41)*	1.30 (1.06, 1.60)*
Male sex (vs female sex)	1.16 (1.04, 1.29)**	1.14 (0.99, 1.31)
Age (per 1 year increase)	1.05 (1.04, 1.05)**	1.03 (1.03, 1.04)**
Period of cancer diagnosis		
1997–1999	1	1
2000–2002	1.11 (0.96, 1.29)	0.92 (0.76, 1.11)
2003–2005	0.82 (0.69, 0.97)*	0.66 (0.53, 0.82)**
2006–2007	0.76 (0.61, 0.97)*	0.60 (0.45, 0.81)**
Stage		
I	1	1
II	1.80 (1.58, 2.06)**	3.03 (2.46, 3.73)**
III	2.66 (2.33, 3.05)**	5.02 (4.07, 6.18)**
Examined lymph nodes ≥10 (vs no)	0.84 (0.71, 1.00)*	0.79 (0.63, 0.98)*
Radiotherapy (vs no)	0.98 (0.88, 1.09)	1.06 (0.92, 1.23)**
SES		
Low	1	1
Medium	0.78 (0.68, 0.88)**	0.77 (0.65, 0.91)**
High	0.75 (0.66, 0.86)**	0.84 (0.70, 1.00)*
Institutionalised	1.35 (1.09, 1.68)**	1.32 (0.98, 1.78)
Hypertension (vs no)	1.12 (1.00, 1.27)	1.00 (0.85, 1.20)
CVD (vs no)	1.23 (1.09, 1.40)**	1.00 (0.84, 1.20)
CVA (vs no)	1.05 (0.81, 1.34)**	0.95 (0.66, 1.36)
Previous cancer (vs no)	1.19 (1.02, 1.38)*	1.00 (0.80, 1.25)
Lung disease (vs no)	1.41 (1.20, 1.66)**	1.37 (1.10, 1.71)*

Values are HR (95% CI)

*p < 0.05

**p < 0.01

Cancer-specific mortality was also increased among colorectal cancer patients with diabetes. Unadjusted analyses showed a small increased risk of cancer-specific mortality in colon cancer patients with diabetes compared with those without diabetes (HR 1.17, 95% CI 1.01, 1.36; data not shown). However, after adjustment for confounding variables, this increased risk disappeared (HR 1.05, 95% CI 0.90, 1.23; Table 4). Bivariate analyses showed that age, sex and receipt of adjuvant therapy were the strongest confounding variables, whereas addition of having had a previous cancer or other comorbidities to the multivariate model did not significantly change the observed HR. We also observed a statistically significant interaction between diabetes and sex for patients with colon cancer. Women with colon cancer and diabetes had an increased risk of colon cancer mortality, whereas men did not. However, after adjustment for age, this finding disappeared.

Cancer-specific mortality was significantly increased among rectal cancer patients with diabetes compared with those without (HR 1.48; 95% CI 1.21, 1.81; data not shown). After adjustment, cancer-specific mortality remained significantly higher for rectal cancer patients with diabetes than for those without diabetes (HR 1.30, 95% CI 1.06, 1.41; Table 5). Again, age and sex appeared to be strong confounding variables, changing the HR by more than 10%, whereas addition of having had a previous cancer or other comorbidities to the multivariate model did not significantly change the observed HR. There was no interaction between diabetes and age, stage, number of lymph nodes examined, receipt of adjuvant therapy, SES and year of diagnosis on (disease-specific) mortality among colon or rectal cancer patients.

## Discussion

This population-based study on more than 10,000 stage I–III colorectal cancer patients reveals that diabetes at the time of rectal cancer diagnosis was associated with an HR of 1.30 for the risk of colorectal cancer mortality compared with those without diabetes. Colon cancer patients with diabetes did not have an increased risk of death from colorectal cancer compared with those without. Furthermore, our results confirm previous findings that overall mortality was increased among patients with pre-existing diabetes at colon or rectal cancer diagnosis.

A previous meta-analysis showed that patients diagnosed with cancer who had pre-existing diabetes were at increased risk of long-term, all-cause mortality compared with those without diabetes [7]. It is, however, possible that the excess mortality risk related to diabetes is completely independent of cancer and cancer treatment. In order to understand whether diabetes influences cancer prognosis above and beyond the prognosis by each disease state independently, one should evaluate disease-specific mortality and adjust for potential differences in cancer stage or treatment between patients with and without diabetes. However, the subsidiary meta-analysis of studies that distinguished between cancer and non-cancer mortality was inconclusive [7], as only a few studies have evaluated disease-specific mortality among cancer patients with diabetes. These studies yielded conflicting results because of a small number of diabetes patients, a limited number of deaths, or no information about cancer treatment [12, 13, 15–18]. Nevertheless, recent analyses of a large prospective cohort showed increased all-cause mortality, colorectal cancer-specific mortality and CVD-specific mortality among colorectal cancer patients with diabetes [14].

In our study we again observed that colon cancer patients with diabetes less often received adjuvant chemotherapy than non-diabetic cancer patients [5, 25]. This apparently also confounded the association between diabetes and cancer mortality among colon cancer patients. This observation is confirmed in a systematic review that evaluated 34 studies on the impact of comorbidity on chemotherapy use and outcomes [26]. Most studies in this review reported lower use of chemotherapy and worse outcomes among cancer patients with comorbidities [27, 28].

For rectal cancer, we did not observe a different stage distribution or less aggressive treatment in patients with concurrent diabetes. Therefore our current findings may imply that the increased disease-specific mortality risk in rectal cancer patients with diabetes follows from a specific interaction between diabetes and cancer.

Several explanations for a worse disease-specific outcome have been discussed in previous papers. It is possible that diabetic patients respond differently to adjuvant therapies. A small retrospective review in rectal cancer patients showed that diabetic patients (*n* = 17) showed higher local progression rates after neoadjuvant chemoradiotherapy than non-diabetic patients (*n* = 93), whereas none of the diabetic patients achieved a pathological complete response [29]. Also, Meyerhardt and colleagues found a significantly higher rate of cancer recurrence and overall mortality among diabetic cancer patients in an adjuvant chemotherapy trial for colon cancer patients, suggesting a smaller effect of therapy [30]. In a review on colorectal cancer outcomes in patients with and without diabetes, an increased risk of cancer recurrence, non-response to chemoradiotherapy and treatment-related complications was reported [8]. However, the authors’ overall conclusion was that much of the long-term mortality risk in colorectal cancer patients with diabetes can be attributed to causes other than cancer.

In our study, we did not observe a significant interaction effect between adjuvant chemotherapy or radiotherapy and diabetes on survival. Unfortunately, as in most studies in this field [26], we did not have information about tolerability or toxicity of treatment in diabetes patients.

Another explanation for the increased mortality risk is increased tumour cell proliferation and metastases in a physiological environment of hyperinsulinaemia and hyperglycaemia, leading to a worse prognosis [31]. Studies among non-diabetic breast and colon cancer patients revealed that high levels of fasting insulin had an adverse prognostic effect on distant recurrence and death [32, 33]. In addition, retrospective, observational studies on diabetic treatment suggest that long-acting insulin glargine (A21Gly,B31Arg,B32Arg human insulin) may increase the risk of cancer [34], whereas biguanide metformin may decrease cancer risk [35, 36] and cancer mortality [37]. Furthermore, obesity and physical inactivity are also associated with disease progression and mortality in non-diabetic colorectal cancer patients [38–43]. The mediators for the elevated risk of colorectal cancer, cancer recurrence and death are not known, but are thought to be related to hyperinsulinaemia, insulin resistance, insulin-like growth factor, adipocytokines and inflammatory cytokines [44, 45]. All these findings suggest that hyperinsulinaemia may stimulate cancer progression, due to its mitogenic effect [46], and support the idea of biological interaction. However, the clinical relevance of the pro-cancer effect of insulin in diabetic patients is still unclear [46].

As we unfortunately did not have information about the diabetes duration and insulin requirement, as well as metabolic control and body mass index, we hope that our planned studies in this field will answer these questions.

Another limitation of this study is the fact that we did not have information about the number of chemotherapy or radiotherapy courses and possible interruption of treatment due to complications in those with or without diabetes. Furthermore, although we believe that a large population-based study that combines cancer stage and treatment variables with cause-specific mortality data is very much needed in this field, the latter also has limitations. Attribution of cause of death is often problematic, especially for colorectal cancer, where records for the UCOD do not accurately specify between colon and rectal cancers [47]. We observed that 2% of patients diagnosed with colon cancer had rectal cancer as the UCOD, whereas 34% of rectal cancer patients had colon cancer as the UCOD. These findings are comparable to a recent US study in which the UCOD records disagreed with California Cancer Registry records for 700 (6%) of 11,404 colon cancer deaths and with 1,958 (39%) of 5,011 rectal cancer deaths, and 82% of the misclassified rectal cancer deaths were coded as colon cancer deaths in the UCOD [47]. Reclassification decreased cause-specific survival for both colon and rectal cancers, but the impact was more pronounced for rectal cancer. Interchangeable use of the terms colon cancer and colorectal cancer is probably one of the reasons for UCOD misclassification. Nevertheless, we believe that by including colon and rectal cancer as the cause of death for both sites, we did not overestimate cause-specific survival. Also, this apparent misclassification is unlikely to have been systematically different for patients with or without diabetes.

Another limitation is the fact that we analysed colorectal cancer death in the presence of competing risks, such as death due to cardiovascular events. As diabetic cancer patients have a higher risk of dying from a cardiovascular event than cancer patients without diabetes, Kaplan–Meier estimates as well as standard Cox regression may have overestimated the actual incidence of cancer death [48]. Therefore it is important not to ignore other causes of death and also include overall mortality analyses to obtain a comprehensive picture of the impact of diabetes on outcomes.

Furthermore, we were not able to include mortality information from the background population without cancer. While most studies among non-cancer populations find standardised mortality ratios of 2–3 for diabetes vs non-diabetes in a similar age-group [49, 50], this study finds much lower HRs for comparisons of diabetes with non-diabetes.

In conclusion, this large population-based study has confirmed previous findings that overall mortality is increased among patients with pre-existing diabetes at diagnosis of colon or rectal cancer. Most interesting was our observation that diabetes at the time of rectal cancer diagnosis was associated with an increased risk of colorectal cancer mortality compared with no diabetes. As this was not explained by differences in stage or treatment of rectal cancer patients with or without diabetes, there might be a specific interaction between diabetes and rectal cancer. We aim to elucidate pathways in future in-depth studies including detailed diabetes- and cancer-related variables.

## Abbreviations

CVA: Cerebrovascular accident
CVD: Cardiovascular disease
ECR: Eindhoven Cancer Registry
SES: Socioeconomic status
UCOD: Underlying cause of death
